# Immune function of colon cancer associated miRNA and target genes

**DOI:** 10.3389/fimmu.2023.1203070

**Published:** 2023-07-03

**Authors:** Lu Han, Shiyun Chen, Zhe Luan, Mengjiao Fan, Yanrong Wang, Gang Sun, Guanghai Dai

**Affiliations:** ^1^ Department of Oncology, The Fifth Medical Center of Chinese PLA General Hospital, Beijing, China; ^2^ Medical School of Chinese PLA, Beijing, China; ^3^ Department of Gastroenterology and Hepatology, The First Medical Center of Chinese PLA General Hospital, Beijing, China

**Keywords:** colon cancer, miRNA, prognosis, immune cell, immune microenvironment

## Abstract

**Introduction:**

Colon cancer is a complex disease that involves intricate interactions between cancer cells and theimmune microenvironment. MicroRNAs (miRNAs) have recently emerged as critical regulators of gene expression in cancer, including colon cancer. There is increasing evidence suggesting that miRNA dysregulation plays a crucial role in modulating the immune microenvironment of intestinal cancer. In particular, miRNAs regulate immune cell activation, differentiation, and function, as well as cytokine and chemokine production in intestinal cancer. It is urgent to fully investigate the potential role of intestinal cancer-related miRNAs in shaping the immune microenvironment.

**Methods:**

Therefore, this paper aims to identify miRNAs that are potentially associated with colon cancer and regulate a large number of genes related to immune function. We explored the role of these genes in colon cancer patient prognosis, immune infiltration, and tumor purity based on data of 174 colon cancer patients though convolutional neural network, survival analysis and multiple analysis tools.

**Results:**

Our findings suggest that miRNA regulated genes play important roles in CD4 memory resting cells, macrophages.M2, and Mast cell activated cells, and they are concentrated in the cytokinecytokine receptor interaction pathway.

**Discussion:**

Our study enhances our understanding of the underlying mechanisms of intestinal cancer and provides new insights into the development of effective therapies. Additionally, identification of miRNA biomarkers could aid in diagnosis and prognosis, as well as guide personalized treatment strategies for patients with intestinal cancer.

## 1 Introduction

Diagnosing colon cancer can be challenging due to the atypical early symptoms, which often go unnoticed by patients, resulting in delayed diagnosis and treatment, poor surgical outcomes, and missed opportunities for surgical intervention (1, 2). Tumor immunotherapy has emerged as a promising new approach for treating colon cancer (3). However, tumor cells can evade immune surveillance and recognition by the immune system, preventing an effective anti-tumor immune response (4). These mechanisms are facilitated by the tumor microenvironment (TME), which is composed of tumor cells, tumor matrix (including tumor-related fibroblasts, immune cells, cytokines, tissue fluid, and neovascularization), and other factors. The TME plays a critical role in the development and progression of tumors, facilitating processes such as malignant transformation, tumor growth, metastasis, and drug resistance (5).

In the tumor microenvironment, certain interstitial cells, including macrophages, adipocytes, and fibroblasts, release extracellular vesicles (EVs), which play a critical role in regulating tumor progression and shaping the microenvironment prior to inflammatory metastasis in the primary tumor microenvironment. Although EVs were initially described as waste products released by cells or carriers for transporting toxins (6), numerous studies have confirmed that they function as important mediators of extracellular signal transduction through direct membrane transfer of their cargo. Uptake of EVs by receptor cells can reprogram signal pathways, thereby regulating the phenotype and function of target cells (7). Small EVs, in particular, have been shown to be involved in various physiological and pathological processes, including cancer development and metastasis. Exosomes, as important participants in intercellular communication and cell “crosstalk,” may act by transferring transmembrane proteins to the plasma membrane to initiate signal transduction, transferring oncogenic proteins, transferring transcriptional regulators to the nucleus to regulate promoter activity, and mediating translation regulation and DNA transfer *via* mRNA/miRNA transfer to integrate into the recipient cell genome. These micro extracellular vesicles play a crucial role in genetic material transmission, intercellular material transfer and transportation, transformation of malignant tumor cells, participation in the tumor microenvironment before and after programmed metastasis, as well as tumor progression, response to treatment, and other processes (8). Exosomes act as natural carriers by transmitting proteins (9), mRNA, or microRNAs to recipient cells. Notably, mRNA and microRNA released from cancer cells or stromal cells can be transferred to nearby cells and maintain their function, affecting the microenvironment of nearby cells. (10, 11). In addition, the secretion of some growth factors, such as TGF- β, EGF, TNF- α And FGF are related to the exocrine membrane (12). Exosomes are also important transmitters of immune responses, such as influencing the interaction between immune cells and antigen presentation (13), and also participating in the process of iron death (14).

Non-coding RNA is not only involved in regulating the expression of carcinogenic or tumor suppressor genes, but also plays a critical role in various tumors, including colon cancer. MicroRNAs (miRNAs) are naturally occurring non-coding small RNA molecules in eukaryotic cells, which affect carcinogenesis, including colon cancer, by post-transcriptionally regulating a variety of oncogenes and tumor suppressor genes. Therefore, understanding the mechanism of miRNAs in tumor development has significant practical implications for the clinical treatment of cancer (15). miRNA can regulate the occurrence and development of tumors by inducing the degradation of genes or inhibiting their protein translation through binding to the complementary regions of 3’-UTR sequences of oncogenes or tumor suppressor genes. For instance, miR-26b overexpression in colon cancer cells can restrain the growth of colon cancer cells and promote apoptosis(16); Similarly, miR-769 targets CDK1 to regulate colon cancer cell proliferation and apoptosis(17), while ectopic expression of -133b leads to G1 phase cell cycle arrest and directly induces colon cancer cell apoptosis (18). Additionally, overexpression of miR-143 in colon cancer induces cancer cell apoptosis. (19). Due to miRNA’s tissue specificity and its ability to regulate gene expression, it may serve as a precise and non-invasive detection method for tumors and become a potential biomarker for early detection and prognosis of colon cancer.

miRNA plays a crucial role in regulating the immune system and various diseases. For example, miR-34a can inhibit Foxp1 to regulate B cell development (20). During lymphocyte development, miR-150 shows a dynamic change in its content, with high expression in mature B and T cells but not in precursor cells. MiR-150 plays a role in lymphocyte differentiation, as its target gene, c-Myb, is an important transcription factor regulating cell differentiation (21). In Crohn’s disease, decreased miR-19b content leads to increased SOCS3 expression, exacerbating the intestinal inflammatory response, but increasing miR-19b content can improve the situation (22). Similarly, miR-193a-3p can alleviate the inflammatory response of the intestinal tract by inhibiting PepT1 in response to intestinal microorganisms (23). Tumor cells release signals to recruit Treg cells to escape the immune system’s elimination, but miR-34a can inhibit CCL22, a vital molecule in the recruitment of Treg cells by liver cancer cells, and increase the infiltration of Treg cells in a mouse liver cancer model (24). In glioma, downregulation of miR-124 is associated with immunosuppressive activity of glioma stem cells (25).

T The Wnt/β-catenin pathway is known to have a pivotal role in the early development of colorectal cancer, with deactivation of the APC gene being a crucial factor leading to the release of β-catenin and activation of the Wnt pathway. This event is considered to be the most significant initiating factor in over 60% of colorectal tumors (26). In colorectal cancer, the levels of miR-135a and miR-135b are upregulated and are inversely correlated with the levels of APC. Studies have shown that miR-135a and miR-135b can directly target and downregulate APC (27, 28), which is a critical event in the early stages of colorectal cancer. In addition, the activation of the EGFR pathway and its downstream effectors KRAS and PI3K can trigger multiple signaling pathways, such as proliferation and angiogenesis, in colorectal cancer. Several miRNAs, including tumor suppressors let-7 and miR-143, can inhibit KRAS, while proto-oncogene miR-21 can inhibit PTEN at the end of the PI3K pathway, and all of these miRNAs are involved in the regulation of these signaling pathways (29). In addition, miRNAs are also involved in TGF-β and P53 pathways in colorectal cancer progression. The involvement of miRNA can affect various aspects of colorectal cancer function. For example, miR-16 has been shown to inhibit the proliferation, migration, and promote apoptosis of colorectal cancer cells by downregulating KRAS. miR-196a can promote the proliferation of colorectal cancer by inhibiting FOXOI and p27Kipl (30). The transcription inhibitor ZEB1 can inhibit the expression of miR-141 and members of the miR-200 family in colorectal cancer. This will relieve the promotion of miR-141 and miR-200c on the differentiation of colorectal cancer epidermal cells, making epidermal cells prone to EMT. The proteins TGF-β2 and ZEB1 that promote EMT have also been shown to be targets for miR-141 and miR-200c, respectively. ZEB1 and miR-200c form a two-way negative feedback to regulate the migration of colorectal cancer. In addition, miR-590-5p can affect the angiogenesis of colorectal cancer by inhibiting the NF90/VEGFA axis, playing a role in suppressing cancer miRNA. (31) found that some miRNAs in colorectal cancer cells at different stages exhibit specific changes in content, including miR-31, miR-96, miR-145, and miR-183. The content of miR-31 gradually increases with the progression of cancer, which can be used as a criterion for judging tumor stage. let-7g and miR-181b have been shown to be important indicators of the efficacy of S-1 based chemotherapy.

To sum up, it will be of great significance to study the expression changes and target genes of miRNA in the occurrence and development of colorectal cancer, reveal the role of miRNA in the process of colorectal cancer, and develop miRNA-centered methods for diagnosis, treatment, and prognosis of colorectal cancer.

## 2 Method

### 2.1 Feature extraction and prediction method of colon cancer-associated miRNAs

To better learn miRNAs-related feature and get better prediction performance, a combination of MLP (Multilayer Perceptron) and CNN (convolutional network) is utilized, named CCNET. MLP employs fully connected layers in each layer, allowing the neural network to learn global feature representations. On the other hand, CNN utilizes local receptive fields and weight sharing mechanisms through convolutional and pooling layers, enabling automatic extraction of local features. MLP can be described as follows:


(1) $$Ouput\;=f(\;\sum^{}\left(w_{j}x+b_{j}\right))$$

where w_j_ is the weight vector associated with the connections from the j-th neuron in the i-1th layer to the i-th layer, x is the input vector, and b_j_ is the bias term associated with the j-th neuron. f(·) is the activation function to introduce non-linearity. And CNN is described as follows:


(2) $$Ouput\left[i\right]\;=f(\;\sum^{}\left(w\left[i-j\right]*x\left[j\right]\right)+b)$$

where y[i] represents the i-th element of the output feature map, b is the bias term, x[j] represents the j-th element of the input sequence, w[i-j] represents the (i-j)-th element of the convolutional kernel.

**Figure 1:**
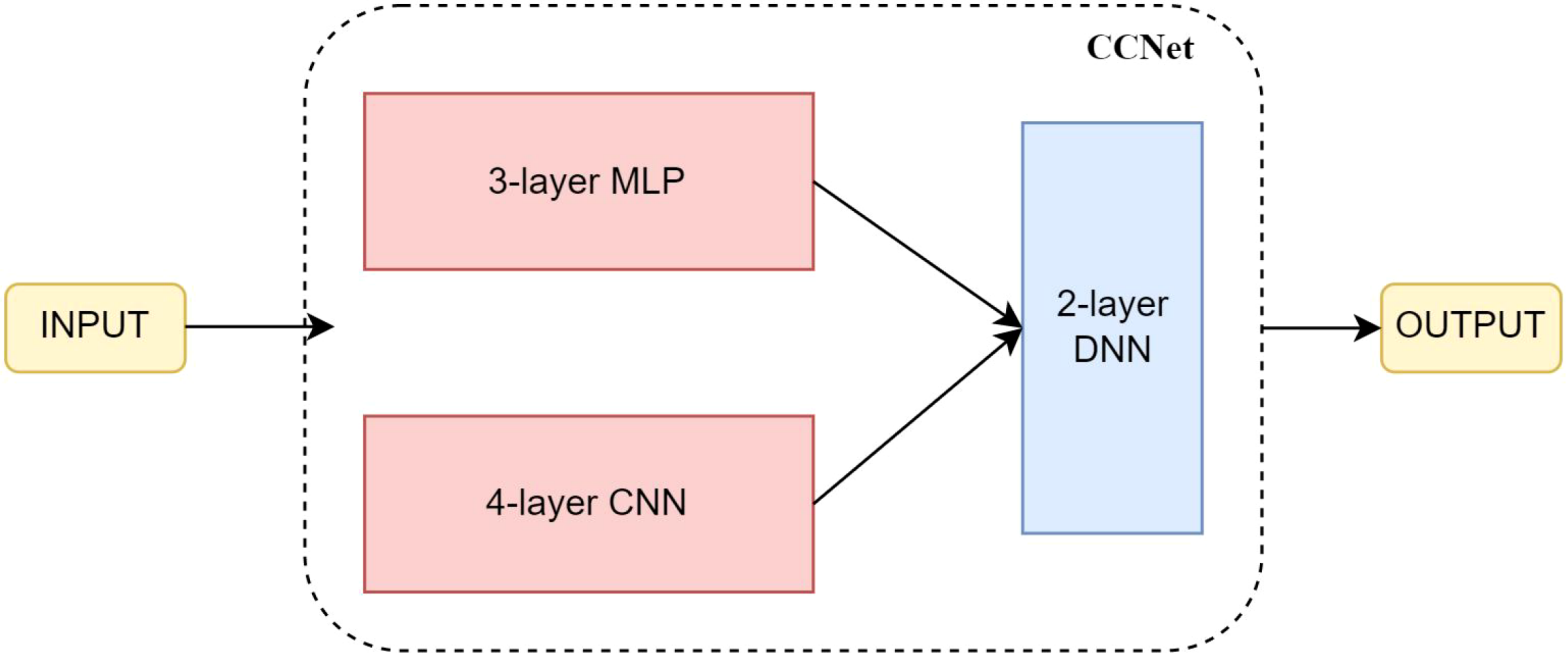
The structure of CCNet.

In the paper, we chose miRNA-related genes as the features in colon cancer. The input features are processed in parallel through two separate branches in the neural network. One branch consists of a 3-layer MLP that focuses on learning global features, while the other branch includes a 4-layer CNN that emphasizes local features. Subsequently, a 2-layer Deep Neural Network (DNN) is employed to fuse and make decisions based on the features learned from both branches. The structure of CCNET as shown in **Figure 1** and **Table 1**.

**Table 1:** The layer details of CCNet.

Module	Layer	Layer Details
3-Layer MLP	1	Fully connected layer (units = 256) ReLu activation function
	2	Fully connected layer (units = 64) ReLu activation function
	3	Fully connected layer (units = 128) ReLu activation function
4-Layer CNN	1	Convolution layer (filters = 256, kernel_size=5) ReLu activation function
	2	Convolution layer (filters = 64, kernel_size=3) ReLu activation function
	3	Convolution layer (filters = 128, kernel_size=3) ReLu activation function
	4	Convolution layer (filters = 128, kernel_size=3) ReLu activation function
DNN	1	Fully connected layer (units = 32) ReLu activation function
	2	Fully connected layer (units = 2)

### 2.2 Survival analysis based on miRNA target immune genes

In survival analysis, LASSO (Least Absolute Shrinkage and Selection Operator) is widely-used in identifying biomarkers, genes, phenotype features and other variables that important on survival time. Its principle is to add an L1 regularization term to limit the size of the regression coefficients, thereby achieving the screening of useless variables and improving the predictive and generalization abilities of the model. LASSO can not only effectively reduce the risk of overfitting, but also select informative variables with a significant impact on survival time. In the paper, we used LASSO to identify key genes in survival analysis of colon cancer.

Then Cox regression model is used to find the optimal model by transforming survival problem into a problem of modeling hazard ratios. The core assumption of the Cox regression model is the proportional hazards assumption, which states that the effects of covariates on hazard ratios are constant over time. This implies that the effects of covariates on hazard ratios are multiplicative and do not vary with time. Based on this assumption, the Cox regression model estimates the regression coefficients of covariates by maximizing the partial likelihood function, obtaining estimates of the effects of covariates on hazard ratios. The Cox proportional hazards model posits that covariates and risks have a multiplicative relationship, and the formula for calculating its risk function is as follows:


(3) $$\text{h}(\text{t}|\text{x})\;={\text{h}}_{0}\left(\text{t}\right)\;*\;\text{exp}\left({\text{α}}_{1}{\text{x}}_{1}+{\text{α}}_{2}{\text{x}}_{2}+\ldots +{\text{α}}_{\text{n}}{\text{x}}_{\text{n}}\right)$$

where h(t|x) represents the conditional hazard function at time t given the covariates x, h_0_(t) is the baseline hazard function, and α_1_, α_2_,…, α_n_ are the regression coefficients corresponding to the covariates x_1_, x_2_,…, x_n_, respectively.

### 2.3 Immune cell infiltration assay method

CIBERSORT (Cell-type Identification By Estimating Relative Subsets Of RNA Transcripts) is a computational method used for quantifying and analyzing the presence and distribution of immune cells within tissue samples, specifically designed for immune cell infiltration assay. CIBERSORT is used by deconvolving the gene expression data of mixed cell populations into the contribution of individual cell types. This is achieved by utilizing a reference gene expression signature for each immune cell type. Then the gene expression profile of the mixed tissue sample is then compared to these reference signatures to estimate the proportions of different immune cell types.

CIBERSORT employs a support vector regression (SVR) model to calculate the cell type proportions, taking into account the potential noise and variability in the gene expression data. It uses a linear model to estimate the proportions of immune cells based on the expression levels of their signature genes, with a regularization step to improve accuracy and robustness.

**Figure 2:**
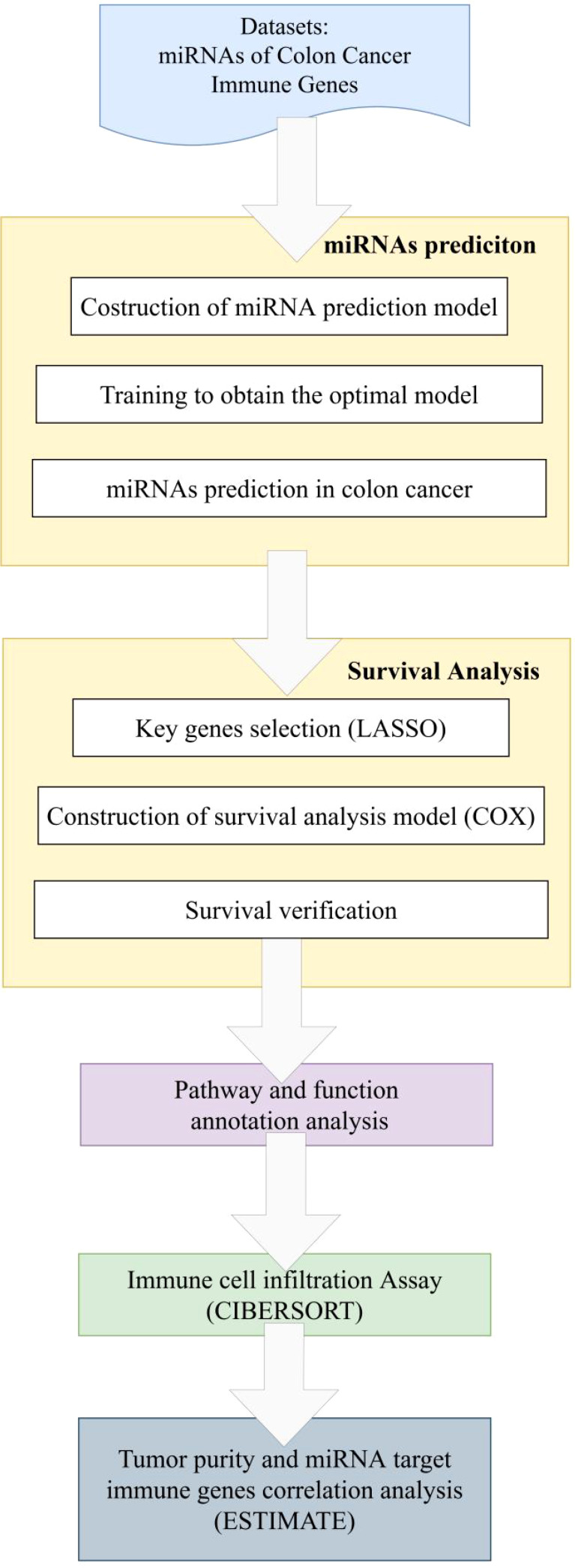
Workflow of immune function studying associated miRNA and target genes.

One of the main advantages of CIBERSORT is its ability to provide quantitative assessment of immune cell infiltration using gene expression data. Additionally, CIBERSORT allows for simultaneous consideration of multiple immune cell subtypes, providing comprehensive information on immune cell infiltration within the tissue sample. Furthermore, CIBERSORT does not rely on prior knowledge of immune cell subtype proportions, making it applicable to various sample types and research designs. The workflow of this paper is shown as **Figure 2**.

## 3 Result

### 3.1 CCNET accurately predicts colon cancer-associated miRNAs

**Figure 3:**
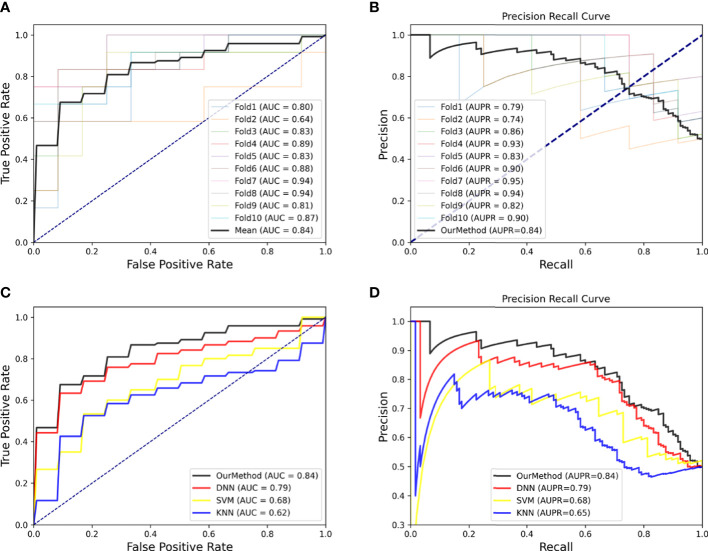
**(A)** ROC curves of 10-cross validation of our method. **(B)** PR-curves of 10-cross validation of our method. **(C)** AUC curves for method comparison. **(D)** AUPR curves for method comparison.

According to biological experiments, 133 miRNAs have been identified as colon cancer-related miRNAs, which we used as positive samples for our model. To generate negative samples, we randomly selected 500 miRNAs with no known relationship to colon cancer. Using these samples, we developed our model and assessed its accuracy in identifying miRNA patterns associated with colon cancer using 10-fold cross-validation to test the AUC and AUPR. **Figures 3A, B** depict the AUC and AUPR curves of each test, along with an average curve for all tests. Furthermore, to demonstrate the superiority of our method, we compared it to other state-of-the-art methods such as Deep Neural Network (DNN), Support Vector Machine (SVM), and K-Nearest Neighbors (KNN), and found that our method achieved significantly higher AUC and AUPR scores, reaching 0.84 (**Figures 3C, D**).

We then used our validated method to input potential colon cancer-associated miRNAs to infer their association with colon cancer. These miRNAs included those related to enteritis, cholecystitis, various cancers, and other digestive diseases, which we believe have potential association with colon cancer due to their association with the disease itself.

### 3.2 Immune genes of colon cancer-related miRNAs

**Figure 4:**
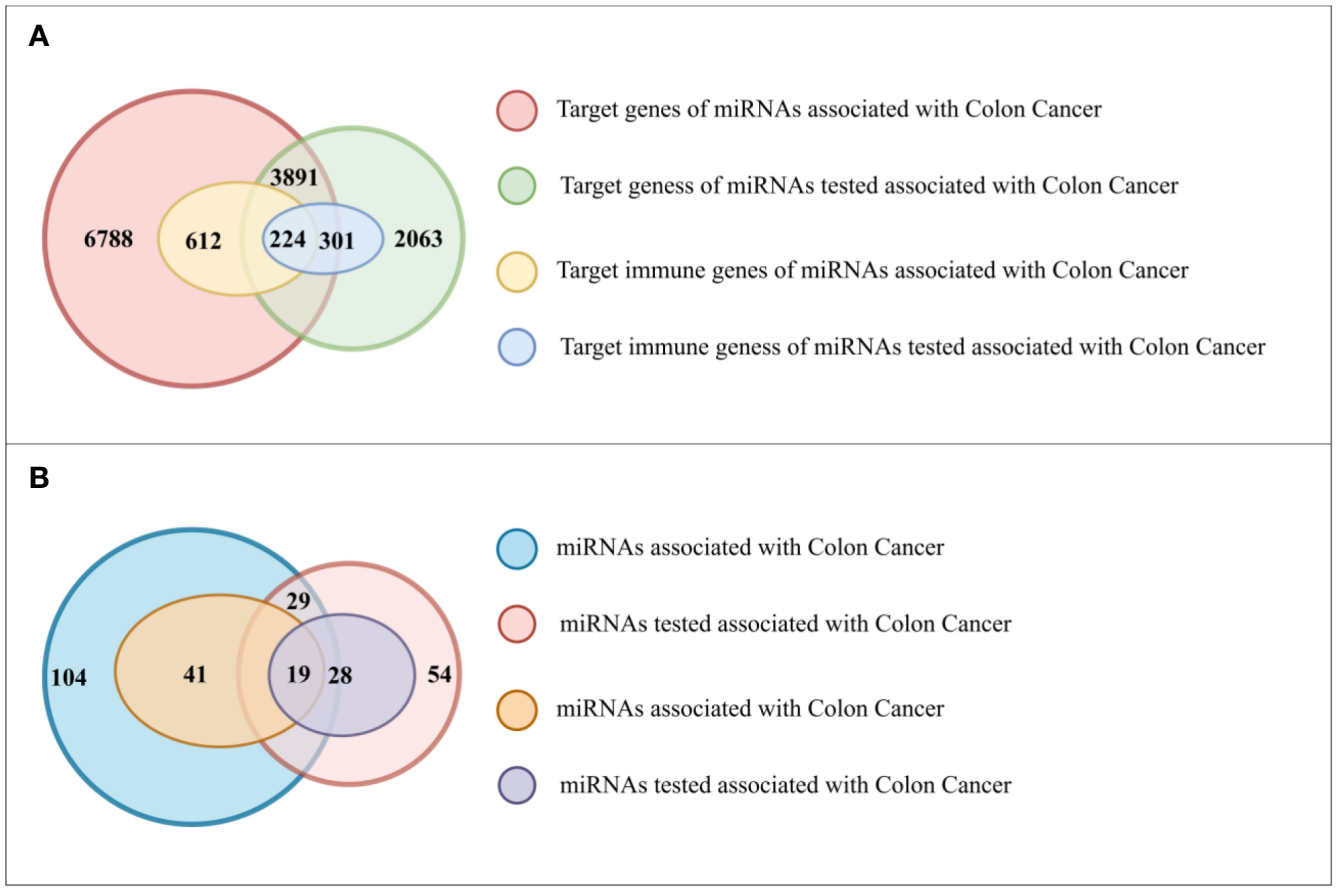
**(A)** The number of genes in different gene sets. **(B)** The number of miRNAs in different gene sets.

Initially, we retrieved immune-related genes from InnateDB, and subsequently collected 133 miRNAs from HMDD, which were reported to be associated with colon cancer. Using miRTarBase as the source of information, we found that these 133 miRNAs had a total of 10,679 target genes, of which 836 were immune-related. Further analysis revealed that 60 of these miRNAs had immune related target genes (**Figure 4A**). We found that 47 of the 83 miRNAs had target genes that were functional in immune responses (**Figure 4B**).

Subsequently, we utilized our method to predict colon cancer-related miRNAs and identified 83 candidates. These miRNAs had a total of 5,954 target genes, of which 525 were immune-related. We found that 47 of the 83 miRNAs had target genes that were functional in immune responses.

### 3.3 miRNA target immune genes plays an important role in the prognosis of colon cancer

**Figure 5:**
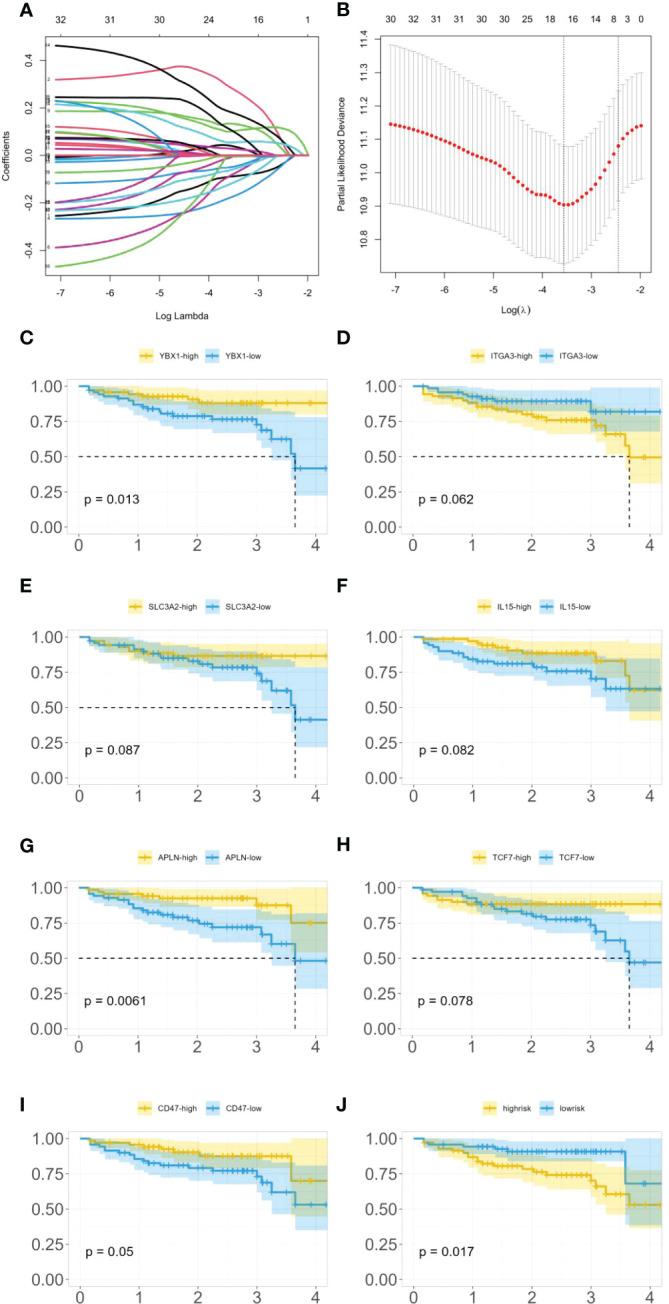
**(A)** LASSO coefficient profiles. **(B)** Tuning parameter (λ) selection in the LASSO model used 10-fold cross-validation. **(C)** Survival curves under different expression levels of YBX1. **(D)** Survival curves under different expression levels of ITGA3. **(E)** Survival curves under different expression levels of SLC3A2. **(F)** Survival curves under different expression levels of IL-15. **(G)** Survival curves under different expression levels of APLN. **(H)** Survival curves under different expression levels of TCF7. **(I)** Survival curves under different expression levels of CD47. **(J)** Survival curves under different risk.

As 525 of these miRNAs target immune genes, we investigated their role in colon cancer prognosis. Using clinical and gene expression data from TCGA, we employed LASSO and Cox regression to determine whether these genes were associated with patients’ survival time as shown in **Figures 5A, B**. After LASSO, we identified seven candidate genes: YBX1, ITGA3, SLC3A2, IL-15, APLN, TCF7, and CD47. **Figures 5C–I** illustrates the differences in survival curves under varying gene expressions. The P-values for these genes were 0.013, 0.062, 0.087, 0.082, 0.0061, 0.078, and 0.05, respectively (as shown in **Figures 5C–I**). Three genes passed the significance threshold: YBX1, APLN, and CD47.

A unique phosphorylation site Ser-176 was found on YBX1 by (32). HEK293 cells and colon cancer HT29 cells that overexpressed the YBX1-S176A (serine to alanine) mutant showed a significant decrease in NF-κB activation ability compared to WT-YBX1-κB, confirming that Ser-176 phosphorylation activates NF-κB for YBX1-κB. Notably, the Ser-176 mutant and the Ser-165 locus each have unique and irreplaceable functions, making them potential targets for cancer treatment strategies. Furthermore, multiple studies have reported on the strong regulation between YBX1 and NF-κB in colon cancer (33, 34).

In a study by (35), the importance of apelin and apelin receptor (APJ) in regulating colon cancer cell motility was examined. The authors found that silencing the APLN gene had an effect on colon cancer cell migration. Additionally, (36) reported that exogenous apelin had anti-apoptotic effects on colon cancer cells.

CD47 binds to a receptor on immune cells called SIRPα, which initiates a signaling pathway that leads to the inhibition of phagocytosis. (37) found that CD47 is overexpressed in colon cancer cells, which can contribute to the immune evasion of these cells. Additionally, they found that the enzyme SHP2 plays a role in regulating CD47 expression and function in colon cancer cells. Through a series of experiments, the authors showed that inhibiting SHP2 activity in colon cancer cells reduced CD47 expression and increased phagocytosis by immune cells. This suggests that targeting the CD47/SIRPα axis may be a potential therapeutic strategy for colon cancer immunotherapy. (38) found that the expression levels of CD47 and SIRPA correlated with poor prognosis and advanced tumor stage. Furthermore, high expression of CD47 and SIRPA was associated with low infiltration of CD8+ T cells and M1 macrophages, which are important effector cells of antitumor immunity. They speculated that targeting the CD47-SIRPA checkpoint might be a potential therapeutic strategy to enhance antitumor immunity in colorectal cancer.

Furthermore, the expression of these genes was classified into high-risk and low-risk categories, and a survival analysis (as shown in **Figure 5J**) demonstrated a statistically significant difference in survival time between these categories with a P-value of 0.017.

### 3.4 Cell infiltration level and Functional analysis of miRNA target immune genes

**Figure 6:**
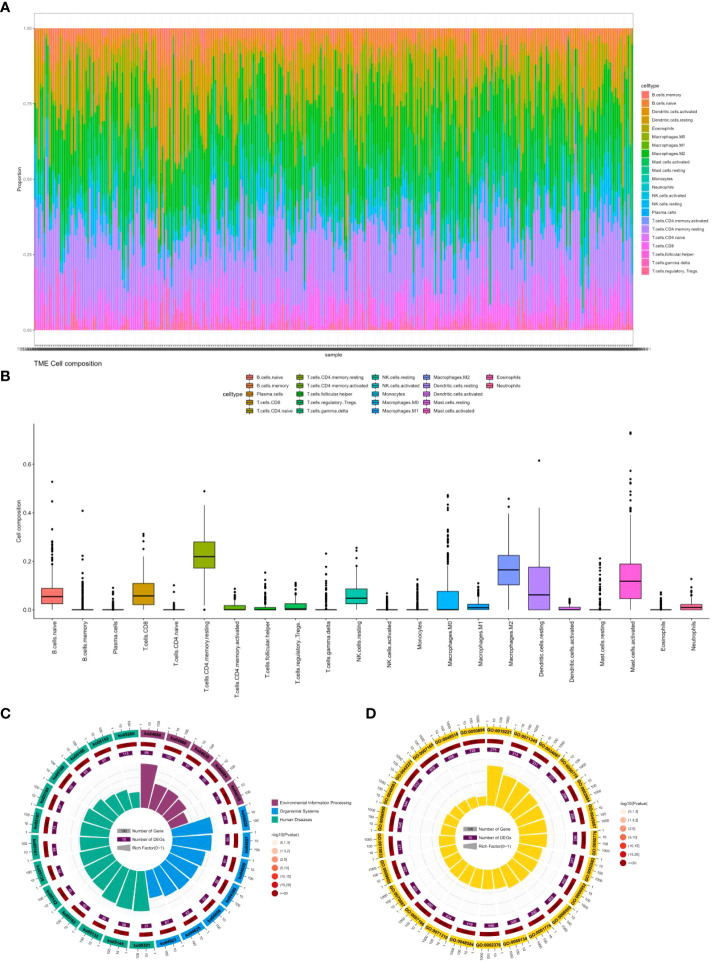
**(A)** Percentage abundance of six types of tumor-infiltrating immune cells in colon cancer. **(B)** Differential abundance of 22 types of tumor-infiltrating immune cells in colon cancer. **(C)** Top 25 KEGG analysis of immune cell marker genes in colon cancer. **(D)** Top 25 GO analysis of immune cell marker genes in colon cancer.

As shown in **Figures 6A, B**, through our analysis of cell infiltration levels of various immune genes, we observed a high abundance of CD4 memory resting cells, macrophages.M2, and Mast.cell.activated cells.

CD4 memory resting cells are a subtype of T lymphocytes that can mount a rapid immune response upon re-exposure to a previously encountered antigen (39). CD4 memory resting cells can play a dual role in both the immune response to cancer cells as well as the development and progression of colon cancer (40). These cells can recognize and attack cancer cells expressing specific antigens through direct cytotoxicity or by releasing cytokines that activate other immune cells and recruit them to the site of the tumor. However, CD4 memory resting cells can also promote the growth and progression of cancer by promoting angiogenesis and remodeling of the extracellular matrix. Studies have found that CD4 memory resting cells are positively associated with the expression of PD-L1 (41), an immune checkpoint molecule that inhibits the anti-tumor immune response, and can have a pro-tumor effect. The role of CD4 memory resting cells in colon cancer is complex and is likely influenced by various factors such as the cancer stage, antigenic profile, and the immune microenvironment(42). In early-stage colon cancer, CD4 memory resting cells may be associated with a better prognosis by contributing to the anti-tumor immune response. In contrast, in advanced-stage colon cancer, the pro-tumor effects of CD4 memory resting cells may outweigh their anti-tumor effects, resulting in a poorer prognosis (43).

Macrophages.M2 are a type of immune cell that plays a role in tissue repair and has anti-inflammatory properties (44). However, in the context of colon cancer, macrophages.M2 cells can promote tumor growth and progression through various mechanisms. They can secrete growth factors and cytokines that stimulate cancer cell proliferation and migration (45). Additionally, they can promote angiogenesis and remodeling of the extracellular matrix, both of which facilitate tumor growth and metastasis. Furthermore, macrophages.M2 cells can inhibit the anti-tumor immune response by producing cytokines and chemokines that recruit other immune cells to the tumor microenvironment, including regulatory T cells and myeloid-derived suppressor cells (46). These cells, in turn, can suppress the activity of cytotoxic T cells and natural killer cells. Additionally, macrophages.M2 cells can express immune checkpoint molecules, such as PD-L1 and CTLA-4 (47), that can inhibit the activity of cytotoxic T cells. The role of macrophages.M2 cells in colon cancer is complex and likely dependent on various factors, including the stage of the cancer, the specific cytokine and chemokine profile of the tumor microenvironment, and the composition of the immune microenvironment. For example, in early-stage colon cancer, the presence of macrophages. M2 cells in the tumor microenvironment may be associated with a better prognosis due to their contribution to tissue repair and anti-inflammatory responses. However, in advanced-stage colon cancer, the pro-tumor effects of macrophages.M2 cells may outweigh their anti-inflammatory effects, leading to a poorer prognosis (48).

Mast.cell.activated cells are a type of immune cell that is involved in allergic reactions and inflammation. The role of mast cell activation in colorectal cancer is not fully understood (49). Several studies have shown that mast cell activation can promote tumor growth and progression by promoting angiogenesis and extracellular matrix remodeling (50). In addition, mast cell activation can promote the recruitment of other immune cells into the tumor microenvironment, such as regulatory T cells and myeloid-derived suppressor cells, which can suppress antitumor immune responses (51). However, other studies have shown that mast cell activation can exert anti-tumor effects by stimulating the activity of cytotoxic T cells and natural killer cells, which recognize and attack cancer cells (52). In addition, mast cell activation can stimulate the production of cytokines and chemokines that recruit other immune cells, such as dendritic cells and macrophages, into the tumor microenvironment, thereby enhancing antitumor immunity reaction (53).

We also explored the function of these genes by KEGG and Gene Ontology. **Figures 6C, D** displays only the top 25 pathways and GO terms. Among them, the most significant pathway is ko04668, which contains 114 genes, including 66 colon cancer-related genes. This pathway, known as the cytokine-cytokine receptor interaction pathway, encompasses various cytokines and their receptors involved in regulating the immune response. In colon cancer patients, the dysregulation of this pathway can occur, contributing to cancer development and progression (54). Alterations in cytokine expression and signaling, resulting from the dysregulation of the ko04668 pathway, can impact the immune microenvironment of the tumor. Studies have found that cytokines and their receptors in this pathway are related to colon cancer progression and prognosis (55). For instance, interleukin-6 (IL-6) and its receptor (IL-6R) are overexpressed in colon cancer tissues, and their expression levels correlate positively with tumor stage and poor prognosis (56). Other cytokines and receptors in this pathway, such as IL-10, tumor necrosis factor (TNF), and transforming growth factor-beta (TGF-β), have also been linked to colon cancer progression and metastasis (57).

The most significant GO term is GO:0019221, which pertains to the cytokine-mediated signaling pathway. This term encompasses the series of molecular events that take place when cytokines bind to receptors on the surface of cells, resulting in the activation of intracellular signaling pathways and eventually changes in gene expression or cellular behavior. This GO term is similar to the ko04668 pathway, which is the most significant pathway we found. Cytokines are crucial regulators of immune responses and have been associated with the development and progression of colon cancer. The dysregulation of cytokine-mediated signaling pathways can contribute to the formation of an immunosuppressive tumor microenvironment, enabling cancer cells to avoid immune surveillance and promoting tumor growth and metastasis. Several cytokines, including interleukin-6 (IL-6), tumor necrosis factor-alpha (TNF-α), and transforming growth factor-beta (TGF-β), have been identified as important regulators of colon cancer development and progression (58). These cytokines can activate various signaling pathways (59), including the JAK/STAT pathway and the NF-κB pathway, both of which have been shown to be dysregulated in colon cancer (60, 61).

## 4 Discussion

We identified 83 intestinal cancer-related miRNAs whose target genes are closely related to immune function. We first found that the expression of three genes regulated by these miRNAs was significantly correlated with the survival time of intestinal cancer, and then found that CD4 memory resting cells, macrophages. M2, and Mast cell activated cells in patients with intestinal cancer were abundant. These three types of cells have been reported to be closely related to the prognosis of colorectal cancer. Through enrichment analysis, we found that the target genes of miRNAs are closely related to the functions of cytokine-cytokine receptor, and cytokine-cytokine receptor is also an important pathway closely related to the occurrence, development and prognosis of colon cancer.

Studies have shown that miRNAs play a critical role in the maintenance of CD4 memory resting cells by regulating the expression of key genes involved in cell cycle progression, survival, and differentiation (62). For example, miR-150 has been shown to regulate the differentiation of CD4 memory T cells into effector T cells by targeting the transcription factor c-Myb (63), which is important for T cell activation and proliferation. miR-146a has been shown to regulate the survival of CD4 memory T cells by targeting the signaling adapter molecule TRAF6, which is involved in the regulation of cell survival and apoptosis (64). Overall, miRNAs play a crucial role in regulating the quiescent state of CD4 memory resting cells and their ability to rapidly respond to antigen re-exposure.

miRNAs are involved in the regulation of M2 macrophage polarization by modulating the expression of genes involved in immune response, metabolism, and tissue repair. For example, miR-155 is known to promote M1 macrophage polarization by targeting the anti-inflammatory cytokine IL-13 (65), while miR-223 is known to promote M2 macrophage polarization by targeting the transcription factor Mef2c (66), which is involved in M1 polarization. Other miRNAs, such as miR-34a, miR-21, and miR-146a, have been shown to regulate M2 macrophage polarization by targeting various signaling pathways involved in inflammation, apoptosis, and tissue repair (67, 68).

miRNAs have also been shown to regulate the expression of genes involved in mast cell activation, degranulation, and cytokine production. For example, miR-155 has been shown to promote mast cell activation and degranulation by targeting the suppressor of cytokine signaling 1 (SOCS1) (69), which is a negative regulator of the JAK/STAT signaling pathway. Conversely, miR-223 has been shown to inhibit mast cell activation and cytokine production by targeting the transcription factor NFAT5 (70), which is involved in the regulation of mast cell activation. Other miRNAs, such as miR-125b, miR-146a, and miR-223, have also been shown to regulate mast cell activation by targeting various signaling pathways involved in inflammation, apoptosis, and cytokine production (71).

Recent studies have shown that miRNAs are involved in the regulation of cytokine and cytokine receptor signaling pathways. For example, miR-146a has been shown to regulate the expression of several cytokines and their receptors, including interleukin-1 receptor-associated kinase 1 (IRAK1) (72) and tumor necrosis factor receptor-associated factor 6 (TRAF6), which are important mediators of the Toll-like receptor (TLR) signaling pathway (72). Another example is miR-155, which is known to regulate the expression of cytokines such as interleukin-6 (IL-6) and interleukin-10 (IL-10), as well as their receptors. In addition, miR-21 has been shown to regulate the expression of the cytokine receptor CD40, which is important for B cell activation and the production of immunoglobulins (73).

In summary, our research suggests that miRNA regulation of genes plays an important role in CD4 memory resting cells, macrophages.M2, and Mast.cell.activated cells and cytokine and cytokine receptor signaling pathways, which makes it closely related to the development and prognosis of colon cancer.

## Data availability statement

The datasets presented in this study can be found in online repositories. The names of the repository/repositories and accession number(s) can be found in the article/supplementary material. Gene expression and clinic factors of colon cancer patients can be downloaded from: https://xenabrowser.net/datapages/?cohort=TCGA%20Colon%20Cancer%20(COAD)&removeHub=https%3A%2F%2Fxena.treehouse.gi.ucsc.edu%3A443.

## Ethics statement

The studies involving human participants were reviewed and approved by ethics committee of Chinese PLA General Hospital. The patients/participants provided their written informed consent to participate in this study.

## Author contributions

LH, SC, and ZL designed the experiments, analyzed the data, and wrote the manuscript. MF and YW analyzed the bioinformatic data. GS and GD provided important ideas. This whole work is guided by LH. All authors contributed to the article and approved the submitted version.
